# Should the citrate used in continuous renal replacement therapy be taken into account as a source of calories?

**DOI:** 10.5935/2965-2774.20230202-en

**Published:** 2023

**Authors:** Lucas Gobetti da Luz, Cassiano Teixeira, Marcelo Filippi

**Affiliations:** 1 Departmet of Nephrology, Hospital Moinhos de Vento - Porto Alegre (RS), Brazil; 2 Department of Clinical Medicine - Hospital Moinhos de Vento - Porto Alegre (RS), Brazil

## To the editor

Acute kidney injury is a prevalent organ dysfunction in intensive care units (ICUs) and often affects critically ill patients. Currently, approximately 13.5% of patients require renal replacement therapy (RRT).^(1)^ Critically ill patients are at a high risk of experiencing hemorrhagic events, and therefore, sodium citrate is the preferred method of anticoagulation in continuous renal replacement therapy (CRRT). Sodium citrate is associated with a longer filter lifespan and reduced bleeding risk.^(1)^

Citrate is administered in the extracorporeal circuit, where it chelates ionized calcium and inhibits thrombin generation. Additionally, is an underestimated source of calories.^(2)^ When given it is as a predilution, the citrate of citrate-calcium complexes is partially removed by the effluent fluid. One milligram of citrate provides approximately 2.5kcal, but its metabolic effect is not easily determined due to variations in cellular activity and other interactions.^(3-5)^ Citrates provide a caloric value of 0.59kcal/mmol (or 2.48kJ/mmol) when metabolized in the Krebs cycle. However, the effective caloric gain from citrate depends on the solution used, infused dose, blood filtration rate, filter type, and amount removed by RRT.^(3)^ Some authors suggest that continuous dialysis using trisodium citrate solution can provide between 200 and 600kcal per day.^(3,4)^ Considering that each mmol of citrate potentially has 592 calories,^(4,5)^ the caloric potential of this source appears to be significant in patients undergoing CRRT who receive high amounts of citrate using various protocols. The substantial caloric intake from citrate cannot be overlooked, even when accounting for citrate filtration during dialysis therapy (estimated at a citrate removal of 20 - 50%).^(3)^Table 1 summarizes the likely caloric yield of citrate in three different strategies of CRRT.

**Table 1:** Estimated caloric delivery by each protocol considering blood flow and losses in dialysis and hemofiltration

Treatment protocol	Blood flow rate (Qb) (mL/minute)	Total caloric delivery (kcal/day)	Caloric delivery considering a loss of 20% (kcal/day)	Caloric delivery considering a loss of 50% (kcal/day)
Author´s Protocol	100	289.8	231.8	144.9
	120	347.8	278.2	173.9
	150	434.7	347.8	217.3
Ci-Ca Protocol®	100	340.9	272.7	170.4
	120	409.1	327.3	204.5
	150	511.4	409.1	255.7
Alabama Protocol®	100	255.7	204.5	127.8
	120	306.8	245.5	153.4
	150	383.6	306.8	191.8

Nutritional imbalance in critically ill and CRRT patients can significantly influence patient outcomes, and neglecting to consider the caloric contributions resulting from continuous dialysis anticoagulation with trisodium citrate means ignoring the precision needed in caloric targets for critically ill patients. Therefore, it is essential to account for this factor in the calculation.

## References

[1] Liu C, Mao Z, Kang H, Hu J, Zhou F. (2016). Regional citrate versus heparin anticoagulation for continuous
renal replacement therapy in critically ill patients: a meta-analysis with
trial sequential analysis of randomized controlled trials. Crit Care.

[2] Jonckheer J, Spapen H, Malbrain ML, Oschima T, De Waele E. (2020). Energy expenditure and caloric targets during continuous renal
replacement therapy under regional citrate anticoagulation. A
viewpoint. Clin Nutr.

[3] Jonckheer J, Demol J, Lanckmans K, Malbrain ML, Spapen H, De Waele E. (2020). MECCIAS trial: metabolic consequences of continuous veno-venous
hemofiltration on indirect calorimetry. Clin Nutr.

[4] Rogers AR, Jenkins B. (2021). Calorie provision from citrate anticoagulation in continuous
renal replacement therapy in critical care. J Intensive Care Soc.

[5] Balik M, Zakharchenko M, Leden P, Otahal M, Hruby J, Polak F (2013). Bioenergetic gain of citrate anticoagulated continuous
hemodiafiltration--a comparison between 2 citrate modalities and
unfractionated heparin. J Crit Care.

